# A gold nanoparticle-protein G electrochemical affinity biosensor for the detection of SARS-CoV-2 antibodies: a surface modification approach

**DOI:** 10.1038/s41598-022-17219-7

**Published:** 2022-07-27

**Authors:** Yeganeh Khaniani, Yuhao Ma, Mahdi Ghadiri, Jie Zeng, David Wishart, Shawn Babiuk, Carmen Charlton, Jamil N. Kanji, Jie Chen

**Affiliations:** 1 Departments of Biological Sciences, University of Alberta, Edmonton, AB T6G 2E8 Canada; 2 Department of Electrical and Computer Engineering, Donadeo Innovation Centre for Engineering, University of Alberta, 11-358, 9211-116 St, Edmonton, AB T6G 2H5 Canada; 3 Department of Computing Sciences, University of Alberta, Edmonton, AB T6G 2E8 Canada; 4 Canadian Food Inspection Agency, National Centre for Foreign Animal Disease, Winnipeg, MB R3E 3M4 Canada; 5 Department of Laboratory Medicine and Pathology, University of Alberta, Edmonton, AB T6G 2B7 Canada; 6 Public Health Laboratory, Alberta Precision Laboratories, Calgary, AB Canada; 7 Li Ka Shing Institute for Virology, University of Alberta, Edmonton, AB Canada; 8 Division of Infectious Diseases, Department of Medicine, Cumming School of Medicine, University of Calgary, Calgary, AB Canada; 9 Department of Pathology and Laboratory Medicine, Cumming School of Medicine, University of Calgary, Calgary, AB Canada; 10 Department of Biomedical Engineering, University of Alberta, Edmonton, Canada

**Keywords:** Sensors and probes, Chemical modification

## Abstract

As COVID-19 waves continue to spread worldwide, demand for a portable, inexpensive and convenient biosensor to determine community immune/infection status is increasing. Here we describe an impedance-based affinity biosensor using Interdigitated Electrode (IDE) arrays to detect antibodies to SARS-CoV-2 in serum. We created the biosensor by functionalizing the IDEs’ surface with abaculaovirus-expressed and purified Spike (S) protein to bind anti-SARS CoV-2antibodies. Gold nanoparticles (GNP) fused to protein G were used to probe for bound antibodies. An ELISA assay using horseradish peroxidase-protein G to probe for bound IgG confirmed that the purified S protein bound a commercial source of anti-SARS-CoV-2 antibodies specifically and bound anti-SARS-CoV-2 antibodies in COVID-19 positive serum. Then we demonstrated that our biosensor could detect anti-SARS-CoV-2 antibodies with 72% sensitivity in 2 h. Using GNP-protein G, the affinity biosensor had increased impedance changes with COVID-19positive serum and minimal or decreased impedance changes with negative serum. This demonstrated that our biosensor could discriminate between COVID-19 positive and negative sera, which were further improved using poly(vinyl alcohol)as a blocking agent.

## Introduction

The coronavirus disease (COVID-19) pandemic is caused by the severe acute respiratory syndrome coronavirus-2 (SARS-CoV-2)^1^. SARS-CoV-2 is extremely contagious. In many cases, the infection is minor or asymptomatic. However, in 20% of the cases, in the elderly, in males and in others with chronic health issues, it can lead to severe COVID-19 disease that requires hospitalization, invasive ventilation or possibly death^2^. Prompt testing and diagnosis are critical for identifying, isolating, and tracing the infected individuals to prevent the spread of COVID-19.

Currently, there are three types of COVID-19 tests available: the viral RNA test, the viral antigen test and the anti-viral antibody test (*aka* serology test). The viral RNA test is the current gold standard for clinical diagnoses of SARS-CoV-2 from nasopharyngeal swabs. The test involves amplifying viral RNA using real time reverse transcriptase polymerase chain reaction (rRT-PCR)^3^. Rapid viral antigen tests help to identify potentially infected, higher-risk individuals outside of laboratory settings. Such tests may be used at home (such as the Ellume COVID-19 Home test)^4^ or at point of care (POC) (such as the Quidel Corporation Quickvue SARS Antigen test)^5^ to screen for those with potentially emerging infections that would be further confirmed using the rRT-PCR test. The third test, the COVID-19 antibody test, is usually performed to determine if an individual has been infected and recovered from COVID-19 infection. The antibody test checks for antibodies developed against SARS-CoV-2 which usually remain in the body for more than 15 days after the onset of symptoms^6^. While the antibody test is not a diagnostic test, it can be used as an analysis tool of SARS-CoV-2 recent or prior infections in populations. Antibody testing can help answer questions on COVID-19 epidemics that are currently largely unknown. These questions include (1) How prevalent are the viral infection among certain populations? Also, what is the proportion of asymptomatic infections? (2) What is the variation of antibody response among patients of different ages, genders, underlying complications, etc.? (3) Is there a correlation between the levels of antibody response and the severity of the disease in patients? (4) What is the longevity and persistence of the antibodies in different populations? (5) Does the presence of antibodies protect against re-infection? To answer these questions, one needs large-scale data which can provide details of the antibody responses of various populations to SARS-CoV-2 infections. Such information could inform decision-making on vaccination strategies and COVID-19 therapeutics.

While there are four major structural proteins of SARS-CoV-2, the spike (S), envelope, membrane, and nucleocapsid (N) proteins^7^, most SARS-CoV-2-infected individuals develop antibodies against the S and N proteins. These antigens are used in COVID-19 clinical serology tests. Currently, enzyme-linked immunosorbent assays (ELISA), with high reproducibility, sensitivity and specificity, are the gold standard of many serology tests, including antibody testing for COVID-19 infections. However, ELISAs are costly, time-consuming, multi-stepped assays that require specialized spectrophotometers or spectrofluorometers and specifically trained and certified technicians to perform these assays. As the COVID-19 pandemic is now in its fourth wave in many vaccinated countries, there is a need to track COVID-19 seroconversion to better understand and control this epidemic. A technical gap must be filled to meet the demands for this large number of antibody POC applications.

Currently, there are several COVID-19 lateral flow immunoassays (LFIA) that have received Food and Drug Administration (FDA) and Emergency Use Authorization (EUA) approval. They detect COVID-19 IgM and/or IgG antibodies from fingerpicks blood samples. Portable LFIAs may be helpful for retrospective diagnostic purposes as well as for sero-epidemiological and vaccine seropositivity studies, but often require two antibodies for detection. Moreover, LFIAs are largely qualitative, not quantitative assays that report the presence of the absence of antibody or antigen. While LFIAs use colored nanoparticles or labels to see the results, label-free electrochemical affinity biosensors for detecting COVID-19 antibodies have been reported. These include a 3D printed paper-based ePAD^8^, an impedance sensing platform^9^, a nickel hydroxide screen printed carbon electrode^10^ and an integrated graphene with gold nanostars nanosensor^11^. All these electrochemical biosensors detect COVID-19 antibodies using immobilized RBD of the S protein^8,9^ or the S protein itself^10^. While label-free biosensors measure binding in real time, they do not offer the amplification or sensitivity of labelled biosensors. In this work, we describe a POC biosensor for COVID-19 antibodies detection that uses gold interdigitated micro-electrode (IDE).

IDEs are portable, highly sensitive and enable robust surface modifications of POC biosensor devices. We used IDE patterned on silicon dioxide substrate. This sensor architecture allows for easy modifications of the sensing surface via silanization of the hydroxyl (OH) groups on the glass surface in between the microelectrode gaps^12^. It also enables electrochemical impedance spectroscopy (EIS), a highly sensitive electrochemical detection method, to indicate the absence or presence of COVID antibodies in the serum samples. More specifically, we created an affinity IDE biosensor to capture the anti-S antibodies in serum samples. We immobilized a baculovirus purified SARS-Cov-2 spike protein on the silica gap between the patterned gold strips. To confer high sensor sensitivity, we used succinic anhydride and 1-ethyl-3-(3-dimethylaminopropyl)carbodiimide (EDC)/ *N*-hydroxysuccinimide (NHS) chemistries to ensure a monolayer of S protein was assembled. Then, gold nanoparticles (GNP) conjugated to protein G (which binds IgG specifically^13^) GNP-proteinG, was employed to probe for the bound anti-S antibodies. When COVID-19 antibodies in serum bound to the spike protein, and the GNP-protein G bound to the IgG, substantial positive impedance change occurred that was not seen with COVID-19 negative serum. Using this S-protein affinity IDE chip biosensor, we validated our device using commercial anti-SARS-CoV-2 antibodies. We then evaluated the performance of the device using COVID-19 confirmed clinical samples. The obtained data were compared with the DiaSorin's LIAISON® and Abbott ARCHITECT ELISA assay as gold standards to evaluate the performance of our IDE nanosensors.

## Results

### Characterization of the gold nanoparticles-protein G conjugate

**Figure 1:**
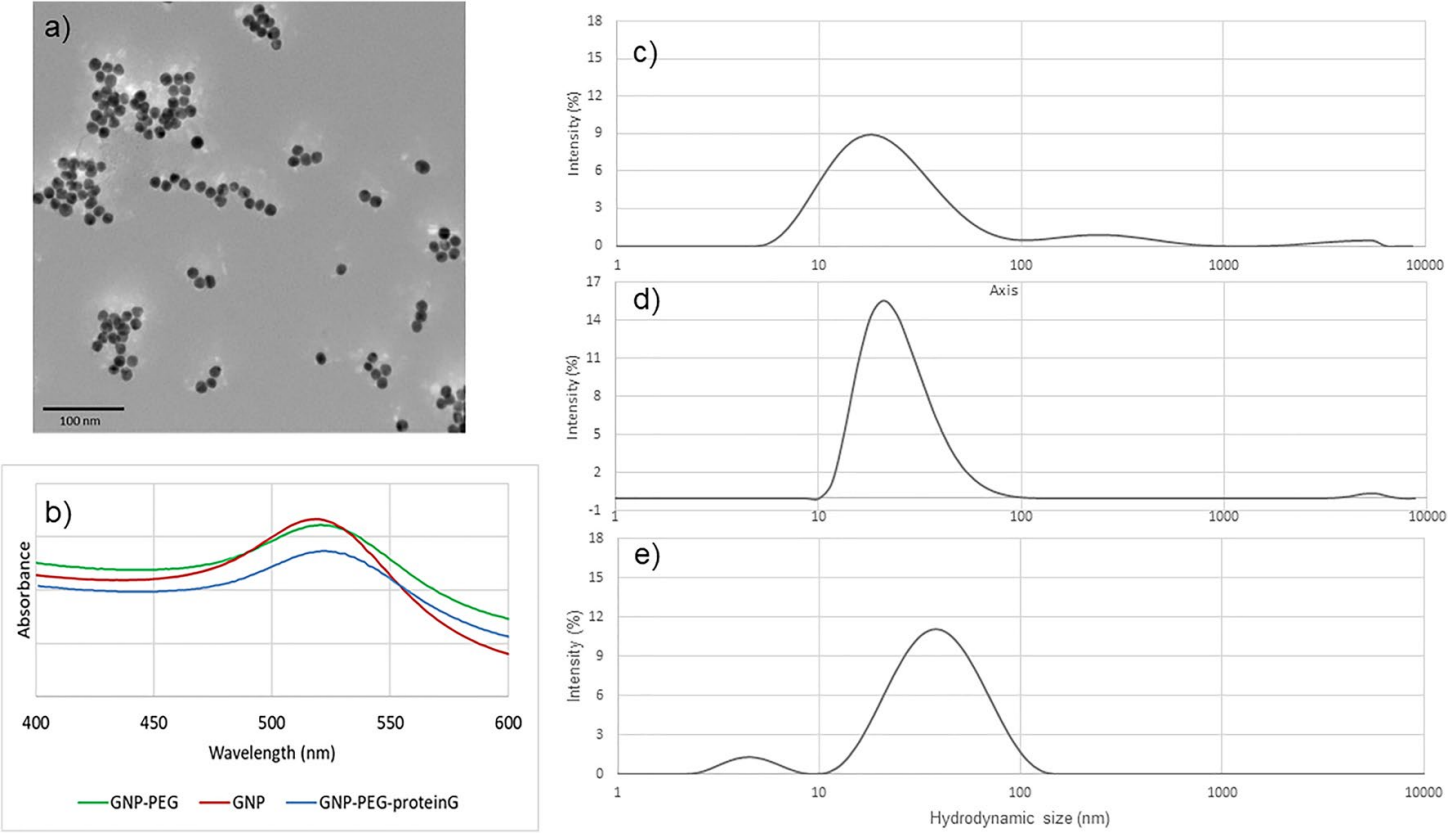
Characterization of the gold nanoparticles (GNP), gold nanoparticles functionalized with polyethylene glycol (GNP-PEG) and affinity labelled with protein G (GNP-PEG-proteinG) (**a**) TEM image of GNPs. (**b**) UV–Vis spectrum of GNP, GNP-PEG and GNP-PEG-proteinG conjugate with λ_max_ = 518, 520 and 521 nm respectively. (**c**) DLS showed the GNP were 18 nm with a zeta potential value − 41 mV. (**d**) DLS showed the GNP-PEG were 35 nm with a zeta potential value − 6.8 mV. (**e**) DLS showed the GNP-PEG-proteinG showed size 37 nm with a zeta potential value − 24.75 mV.

To determine that the GNPs were coated with protein G, the gold nanoparticles with and without functionalization using SH-polyethyleneglycol(PEG)_5000_-COOH and then affinity-labelled with protein G, were quantified by ICP-MS, characterized by ultraviolet–visible spectroscopy (UV–Vis), Transmission electron microscopy (TEM) and dynamic light scattering (DLS) and for colloidal stability by electrophoretic light scattering (ELS). For citrate-capped, unfunctionalized GNPs, the UV–Vis spectrum showed a localized surface plasmon resonance (LSPR) band with an absorbance maximum at 519 nm. Using UV–Vis spectrum data (Abs_LSPR_ and Abs_450_), this batch of GNPs had an average size of 11 nm^14^. The TEM analysis also confirmed that the particle size of the GNPs ranged from 10 to 12 nm (see Fig. 1a). The concentration of gold element measured using ICP-MS was 58 µM. By DLS, the hydrodynamic size data showed a Z-Average of 18 nm (Fig. 1c). According to ELS, the zeta-potential (ζ-potential) value was − 41.6 mV, indicating high colloidal stability. After functionalization of the GNP with SH-PEG_5000_-COOH, the absorbance maximum shifted to 520 nm (Fig. 1b). The distribution of the hydrodynamic diameter increased to a mean of 35 nm (Fig. 1d). This is expected since the SH-PEG_5000_-COOH chain contains 110 PEG units. Conjugation of protein G into the GNP caused another 2 nm shift in UV–Vis absorbance maximum peak from 520 to 522 nm (Fig. 1b). Using DLS, the GNP-protein G had a bimodal distribution with a greater fraction of larger particles with a mean hydrodynamic diameter/size of 37 nm (Fig. 1e) and a zeta-potential value of − 24.75 mV. This bimodal distribution is expected because concentrated samples cause multiple light scattering during the DLS measurement^15^. Thus, using UV–Vis and DLS, we determined that the GNP were functionalized with PEG and then protein G as there were shifts in the UV–Vis in each step (519–520–522 nm) and observed expected increases in the hydrodynamic diameters by DLS (18–35–37 nm). The ζ-potential changed from − 41.6 mV for GNP alone, covered by citrate ions, to − 6.8 mV for GNP-PEG, due to replacement of the citrate anions by neutral PEG units, and − 24.75 mV for the GNP-protein G. Protein G has 12 polar sites on its structure and therefore increase the colloidal stability when conjugate with GNP^16^. The concentration of gold in GNP-protein G determined by ion-coupled plasma mass spectrometry (ICP-MS) was 7.5 mM, much higher than the GNP because the functionalized GNP-PEG was concentrated before conjugation with protein G.

### Determining the optimal serum dilution for detection of anti-SARS-CoV-2-S antibodies using ELISA

**Figure 2:**
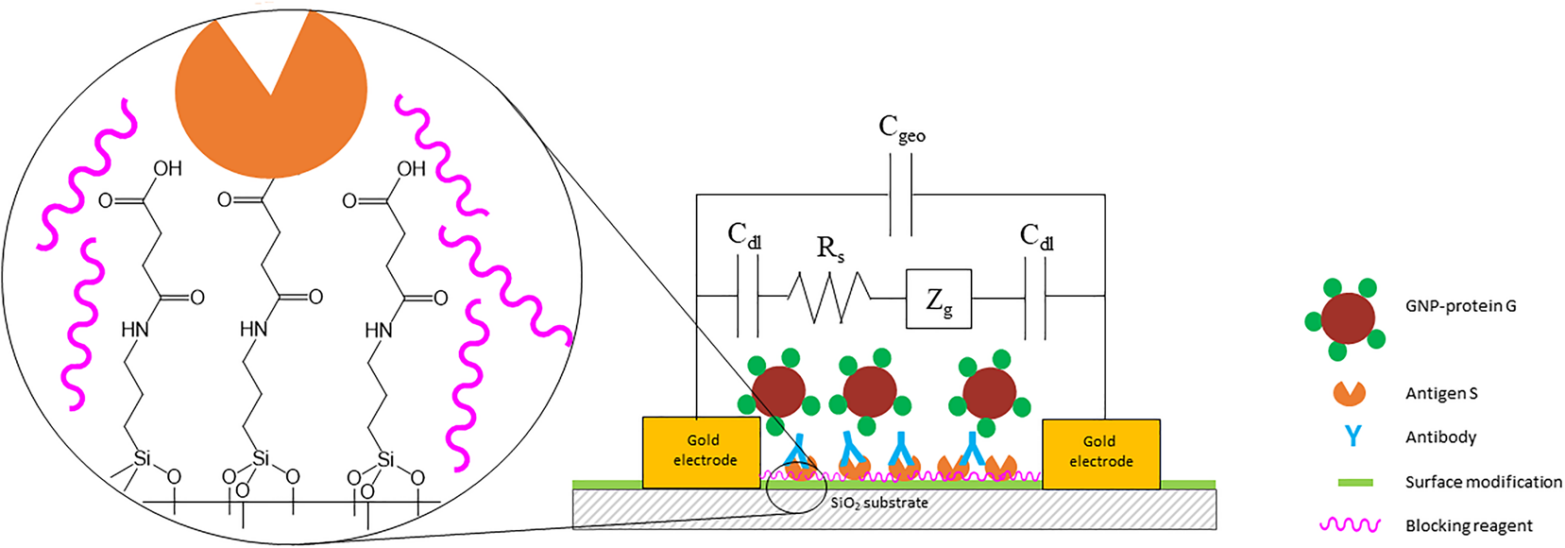
Schematic diagram of the affinity biosensor. Baculovirus purified S protein (antigen S) is immobilized in the gap between interdigitated electrodes. Serum antibodies to the S protein bind to the S protein. Gold nanoparticles conjugated with protein G (GNP-proteinG) bind to the antibodies, causing an impedance change. The zoomed-in area shows the silica gap between gold electrodes with surface modification details, the spike protein immobilization and the presence of blocking agents to improve impedance readings.

Our IDE sensor (schematic diagram shown in Fig. 2) mimics an indirect ELISA assay: an antigen (the viral S proteins) is bound to the plate, antibodies in serum bind to the antigen and the binding of the antibodies is detected with an additional amplification step. In our biosensor, we bound the S protein to the IDEs (Fig. 2). Next, the anti-SARS-CoV-2-S antibodies, either commercially available or in human serum samples, were bound to the S protein. Then the GNP-conjugated protein G (that binds IgG isotype specifically) or the horse radish peroxidae (HRP)-conjugated protein G for ELISAs, binds to the anti-SARS-CoV-2-S antibodies, and amplifies the signal of the bound anti-SARS-CoV-2-S antibodies. For our biosensor, binding of the GNP-protein G causes an impedance change. For ELISAs, the binding of the HRP-protein G to antibodies is detected by adding an oxidizable chromogen or fluorophore, producing a signal that is proportional to the amount of bound antibodies.

**Figure 3:**
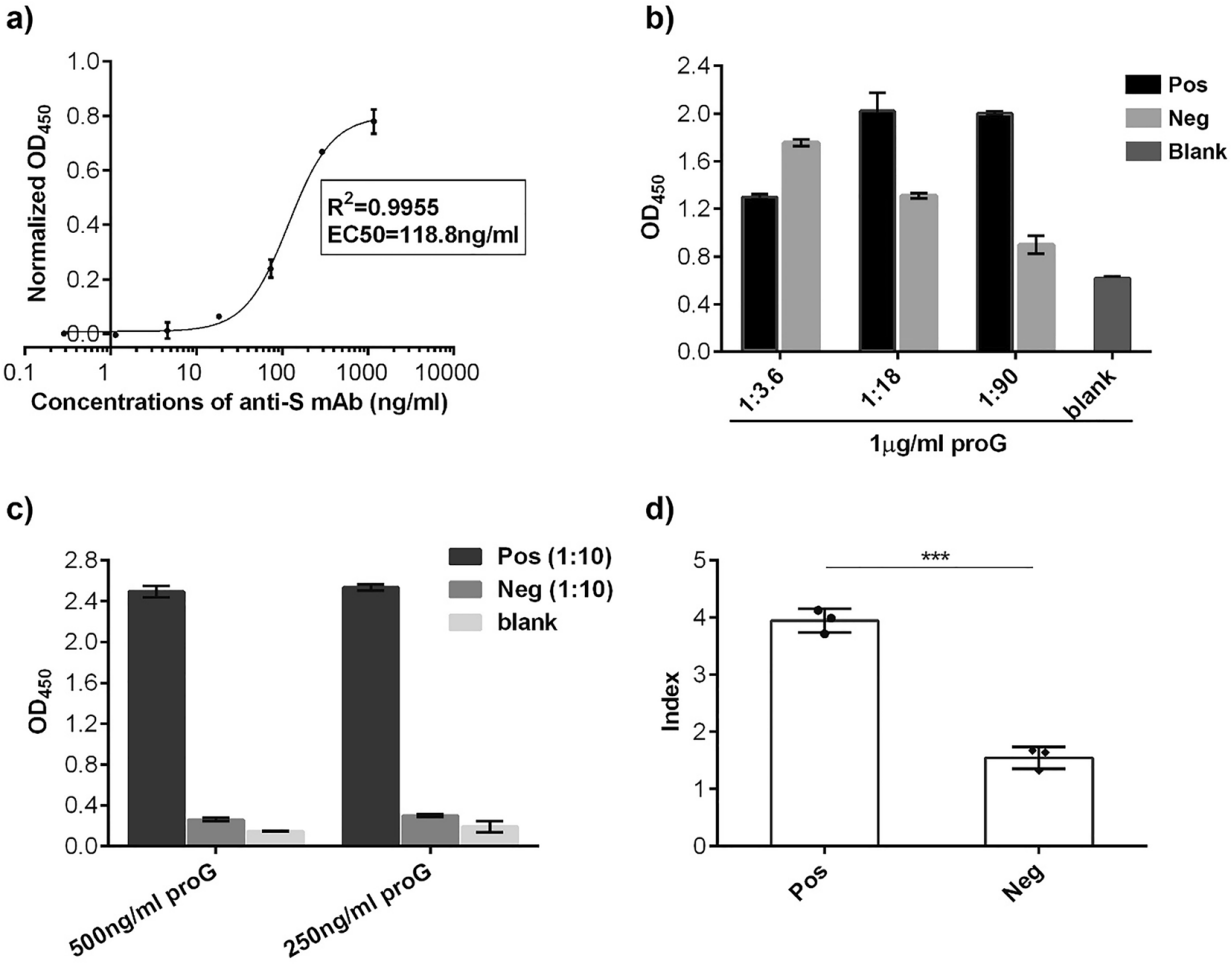
Verifying the assay specificity in detection of anti-SARS-CoV-2-S antibodies (IgG isotype) via ELISA. (**a**) The purified anti-SARS-CoV-2-S monoclonal antibodies (mAb) were detected in ELISA at various concentrations. (**b**,**c**) Anti-SARS-CoV-2-S antibodies (IgG isotype) in human serum samples were detected with serum diluted in (**b**) 1:3.6, 1:18 and 1:90 and in (**c**) 1:10. 1 µg/mL of -HRP protein G was used in (**b**) for detection while 500 ng/mL and 250 ng/mL were tested in (**c**). (**d**) Using the detection scheme for three positive serums and three negative serums could be significantly separated based on the index where index = Sample_O.D.(450 nm)_/blank_O.D.(450 nm)_. ***p < 0.001. Error bars represent the standard deviation.

For our biosensor, we wanted to confirm that our baculovirus purified S protein bound anti-SARS-CoV-2-S antibodies specifically. To confirm the specificity of binding, we employed an ELISA assay using a commercially available anti-SARS-CoV-2-S antibodies (Fig. 3a). When increasing amounts of anti-S antibody were added, increasing amounts acidified 3,3',5,5'-tetramethylbenzidine (TMB) were detected. When the absorbance of TMB was plotted against increasing amounts of commercial antibody, a four-parameter logistic (4-PL) regression curve^17^ was generated (Fig. 3a), which is expected for ELISA assays. R^2^ was 0.9955, indicating a good fitting of the experimental data. The EC50 was 118.8 ± 1.07 ng/mL with a maximum plateau of 1.2 µg/mL. These results indicated that as little as ~ 100 ng/mL of commercial anti-S antibodies bound to our baculovirus purified S protein.

We next tested binding of anti-SARS-CoV-2-S antibodies from a patient’s serum to the baculovirus purified S protein using the ELISA assay. Because false positives occur frequently with serum samples, and because no currently known blocking agents can completely prevent these false positives^18^, we chose to dilute the serum samples to minimize background binding. Background binding can be caused by hydrophobic binding of immunoglobulin components in serum to solid surfaces or from non-specific binding of serum components to the antigen (S protein in our study). We wanted to dilute a negative serum (Neg) sufficiently to achieve absorbance reading approximating the blank while still detecting a signal with the positive serum (Pos). We diluted one Pos and one Neg serum 1/3.6, 1/18 and 1/90 with 0.05% PBST containing 1% milk. As seen in Fig. 3b, there was no separation between Pos and Neg with 1/3.6 diluted serum. However, with 1/18 and 1/90 diluted Pos and Neg, the Neg background signal decreased while the Pos absorbance increased. However, we observed that the blank sample (no serum added) has a high background absorbance (0.570 ± 0.011). This background noise could have arisen from non-specific binding of excess HRP-protein G to the plates. We reduced the HRP-protein G from 1 µg/mL to 500 ng/mL and 250 ng/ml, respectively (Fig. 3c). With 500 ng/mL HRP-protein G, the blank absorbance was reduced to 0.100 ± 0.002 while with 250 ng/mL, the blank absorbance was higher. As 1:90 serum dilution may result in false negatives from weakly positive serum samples, we chose to dilute the remaining serum samples by 1:10. We confirmed that at 1:10 dilution of the Neg sample detected using 500 ng/mL HRP-protein G yielded a minimal background absorbance (Fig. 3b,c). We then tested the three Pos and Neg samples and saw a significant separation between the Pos and Neg samples using the index, where index = Sample_O.D.(450 nm)_/blank_O.D.(450 nm)_ (Fig. 3d).

### Characterization of the biosensor interface after immobilization of spike protein and probing with gold nanoparticles-protein G

Using an indirect ELISA assay, we demonstrated that our baculovirus purified S-protein was recognized by the commercial anti-SARS CoV-2 antibodies and bound antibodies in COVID-2 positive but not negative sera. We also demonstrated that the HRP-protein G bound to the anti-SARS CoV-2 antibodies, yielding the 4-PL regression curve expected for ELISA assays. Therefore, we were confident that we could create an impedance-based affinity biosensor to detect anti-SARS CoV-2 antibodies using the baculovirus purified S-protein and protein G.

Two steps are required to build an affinity IDE sensitive to anti-SARS CoV-2 antibodies. First, after cleaning the IDEs, a self-assembled monolayer (SAM) of carboxy aliphatic compounds must be built. Second, the S protein must be anchored to that SAM. The monolayer was built by incubating the chips with (3-aminopropyl)triethoxysilane and succinic anhydride followed by treatment with EDC/NHS. EDC/NHS activates the carboxylic groups to allow coupling with the amine groups terminating the S protein. We wanted to determine if the S protein was immobilized on the electrode surface. Ellipsometry was used to measure the thickness of S protein in the IDE gap region. The measurement was performed using S protein immobilized on the chip without an electrode since the electrode would interfere with the measurements. Using ellipsometry, we demonstrated that a film of S protein was present on the surface. To determine if the incubation time in the succinic anhydride solution affected the S protein immobilization, we chose three different incubation times: 1, 4 and 19 h. With shorter incubation times, 1 h and 4 h, 6.5 nm and 5.7 nm thick films, respectively, were formed (Fig. S1), with a large variation of thickness (> 1.5 nm). With a much longer incubation time, 19 h, the film thickness increased to 9.0 nm, with a much smaller variation of thickness (0.5 nm). We thus chose 19 h incubation with succinic anhydride. We also tested the immobilization of the S protein using surface amine linkages rather than the carboxyl linkages described above. Catalysts were added to the S-protein to activate the protein carboxylic groups before reacting with the primary amines on the surface. When we tested negative and positive sera binding to the amine-functionalized protein and measured the impedance changes upon GNP-protein G binding, inconsistent results were observed (data not shown). This may have resulted from cross linking of amine and carboxylic acid groups within the S protein, altering the epitopes recognized by the anti-SARS-CoV-2 antibodies. Thus, immobilization of the S protein terminal amines through carboxyl linkages on the surface was the better means to covalently bind the protein to the electrode gap.

We wanted to determine that the GNP-protein G was binding to the antibodies present in the COVID-19 positive serum. AFM was performed to confirm the successful binding of GNP-G to biosensor surfaces incubated with serum from COVID-19 patients. Since the anti-SARS CoV-19 antibodies are present only in COVID positive serum, and absent in COVID negative serum, the GNP-protein G binding should only be detectable in biosensors treated with COVID positive serum. As shown in Fig. S2a, several raised areas with an average height of 20 nm were found on the electrode gap surface. In contrast, fewer raised areas were seen when the electrode surface was treated with COVID-19 negative serum (Fig. S2b). Together, we demonstrated using ellipsometry that the S-protein was immobilized on the biosensor surface and that GNP-protein G was binding to the antibodies known to be present in COVID-19 serum.

### Mechanism of the designed electrochemical biosensor

Electrochemical biosensors generally work based on the application of a small amplitude AC voltage to the electrode and measurement of the phase/current response as a function of frequency. The changes in the electrical properties come from interactions between biological molecules attached to the sensor (spike protein) and an analyte present in the sample (antibodies in this case). It is a non-faradic EIS with no redox component in the system. In non-faradaic approach, the charge is associated with movement of electrolyte ions, reorientation of solvent dipoles, adsorption/desorption, etc. at the electrode–electrolyte interface. The dielectric constant of the substrate material and the morphology of self-assembled monolayer on the electrode affect the sensitivity of the sensor and are evaluated in our group previously^19^.

Gold electrodes are micro-interdigitated on a silica substrate and the gap between the gold digits is decorated with viral S proteins to detect the anti-S antibody. Our developed sensor device relied on the double layer surrounding the nanoparticles generated by the presence of gold nanoparticles in between positive and negative electrodes to result in a measurable impedance change^20^. This phenomenon is called double layer capacitance. Gold nanoparticles provide a capacitive change when bound to the surface between electrodes^21^.

### Validation of affinity biosensor using impedance data analysis

**Figure 4:**
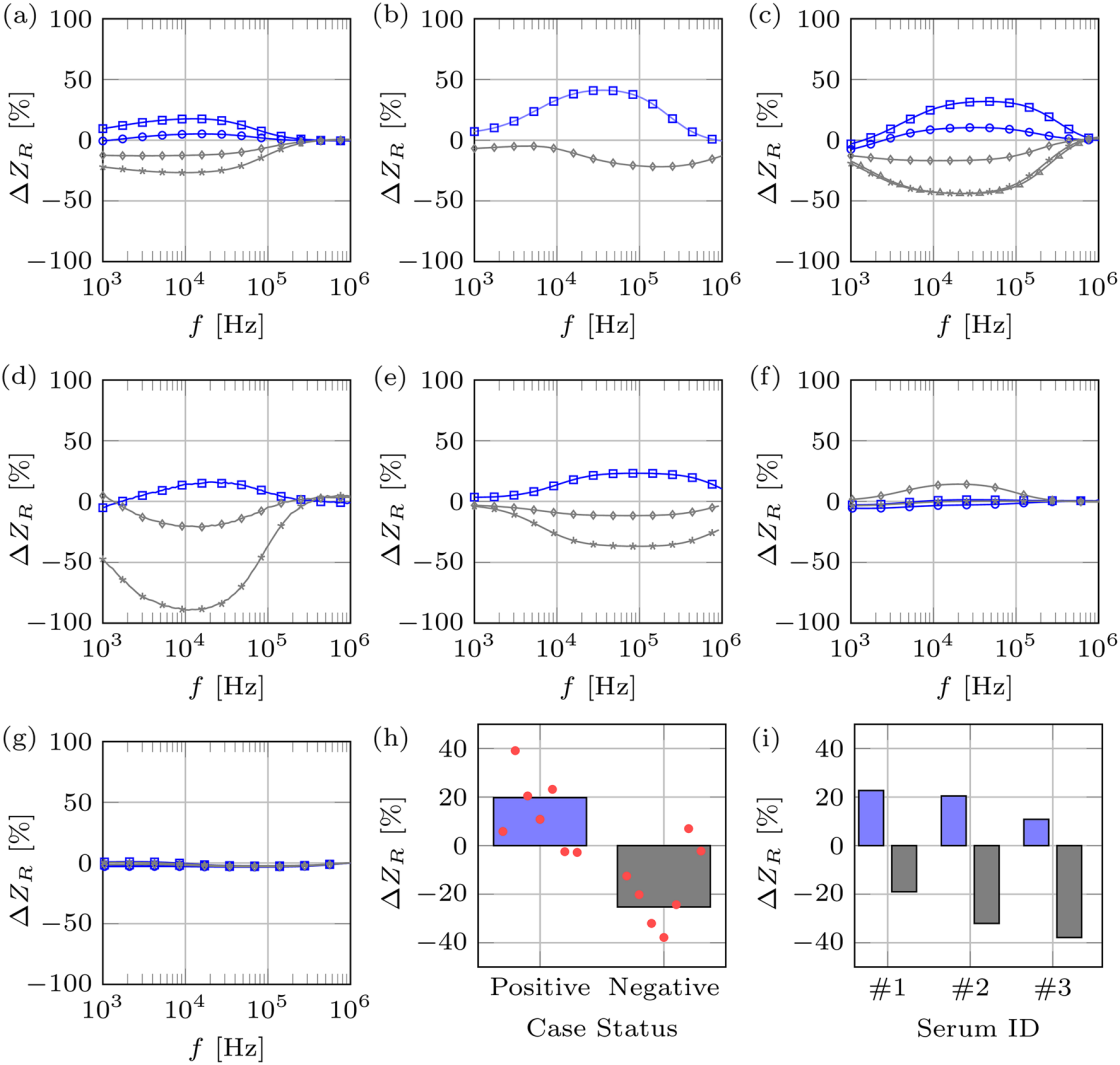
Impedance data collected from affinity biosensor chips after incubation with COVID-19 positive or negative sera followed by binding with GNP-protein G. (**a**–**e**) Impedance data collected from 5 different chips. The blue color indicates positive cases while the gray one indicates negative ones. Each curve corresponds to data collected from a single test. (**f**,**g**) two examples of failed tests, not able to distinguish between positive and negative sera. (**h**)The impedance data, collected from positive or negative sera, tested on the same chips, was averaged and plotted as single points. (**i**) The impedance data collected from individual COVID-19 positive or negative sera from different chips, was averaged.

To demonstrate that our affinity biosensor was able to differentiate the binding of SARS-CoV-2 antibodies, we used impedance measurements. When GNPs bind to electrodes, we expect an increase in the measured impedance. With COVID-19 recovered positive serum, we expect that the GNP-protein G will bind to anti-SARS CoV-2 IgG antibodies, yielding an increase in impedance while no or minimal impedance measurements are expected with negative serum or blank electrodes^20^. Impedance measurements were done after every single step including the surface modification, addition of serum, and the GNP-protein G treatment on washed electrode in diluted PBS buffer. The reported relative impedance data (∆Z_R_) was calculated by subtracting the value of the impedance at the previous step from the current one and dividing by the value of the former. In Fig. 4, the ∆Z_R_ are derived from the last step, the addition of the serum, then the GNP-protein G to the chips. Our affinity biosensor can distinguish between positive and negative sera with notable accuracy (see Fig. 4a–e; each graph represents an individual chip). The positive cases lead to a local maximum in the frequency range of 10^4^–10^5^ Hz, whereas the negative cases (and the blank ones, without serum), lead to a local minimum. When comparing the ∆Z_R_ at a given frequency, the difference between positives and negatives is more pronounced. At around 50 kHz, not only the positive and negatives cases correspond to ∆Z_R_ with the same sign, but also this difference is large enough that can be detected easily.

It’s been shown that there is negligible nonspecific adsorption of nanoparticles on the gold digits surface because of the precipitation of serum components during the incubation time^22^. This adsorbed layer on the gold electrode will bind into the GNP-protein G nonspecifically during the later step of incubation with GNP-protein G. Mentioned nonspecific binding happens in both positive and negative serum samples more or less depending on proteins present in each individual sample and legitimate decrease of impedance in negative samples.

To determine if there was an average frequency that impedance changes were occurring for positive or negative sera within chips, we averaged the ∆Z_R_ from electrodes incubated with positive or negative sera reacted on each chip. The results from each chip’s positive or negative sera (red dots) are shown in Fig. 4f where all positive sera led to positive impedance changes and the negative sera led to negative impedance changes. The ∆Z_R_ from all chips were averaged to determine if there were consistent impedance changes with positive and negative sera. The positive sera yielded an average 19.87% difference (blue bar) and the negative sera yielded -25.37% difference (grey bars), showing that impedance measurements (∆Z_R_ > 0) could reliably distinguish COVID-19 positive from negative sera (Fig. 4f).

To determine if the differences between individual sera (#1, #2 and #3) could be distinguished on different chips, the impedance data collected from each individual positive or negative serum reacted on different chips were averaged and plotted (Fig. 4g). Individual positive serum again yielded positive impedance changes and individual negative serum yielded negative impedance changes. This suggests that our in-house fabricated chips consistently distinguished COVID-19 positive sera from negative sera. Moreover, a ∆Z_R_ = 0 can be used as the threshold to classify the samples into positive or negative cases with an expected impedance change averaging a positive 20% difference for COVID-19 positive cases and an average of negative 25% difference for negative sera when measured at the 10^4^–10^5^ Hz frequency range.

**Figure 5:**
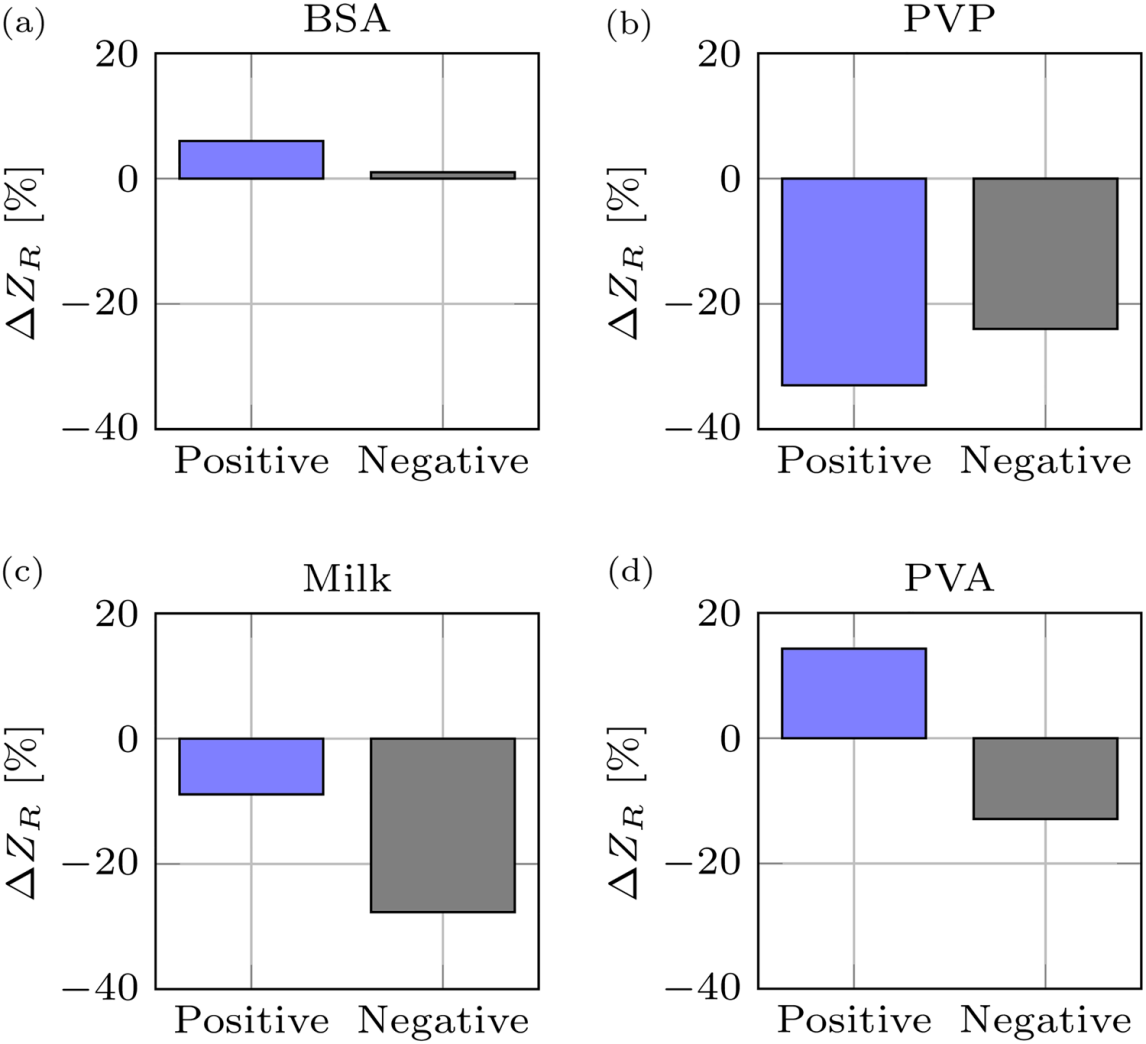
Optimization of impedance signal using different blocking agents. Individual positive and negative serum samples were mixed with (**a**) 1% bovine serum albumin (BSA); (**b**) 3% polyvinyl pyrrolidone (PVP)-10; (**c**) 3% skim milk; and (**d**) 1% polyvinyl alcohol (PVA)-61,000 in PBS buffer.

We further optimized our affinity biosensor to bind anti-SARS-CoV-2 antibodies using different blocking agents. Blocking agents bind to unoccupied spots on the surface, enabling anti-SARS-CoV-2 antibodies to specifically bind to the immobilized S protein rather than non-specifically binding to the other areas of the sensor. Neutral antibodies in serum samples may react and bind to blocking agents such as BSA and casein, yielding a false positive signal in the sensor. We assessed the performance of the biosensor by blocking any unoccupied sites on the S-protein functionalized biosensor with common blocking reagents used with ELISA assays—BSA and skim milk and synthetic compounds—polyvinyl pyrrolidone (PVP) and polyvinyl alcohol (PVA). Data analysis revealed the best blocking reagent was PVA which yielded a positive change in relative Z for positive sample and a negative change in negative serum samples (Fig. 5). The benefits of using PVA as a blocking agent in similarly designed devices were recently reported^23^.

## Discussion

As COVID-19 waves continue to surge worldwide, with breakthrough infections in vaccinated individuals, demand for a portable, inexpensive, and convenient biosensor to determine community immune/infection status is increasing. We developed an in-house impedance-based affinity biosensor using IDE arrays to detect antibodies to SARS-CoV-2 in sera. We created the affinity biosensor by functionalizing the IDEs’ surface with a baculaovirus-expressed and purified Spike (S) protein to bind anti-SARS CoV-2 antibodies from serum. Gold-nanoparticles (GNP) fused to protein G were used to probe for bound antibodies. Using the optimal concentrations developed with the ELISA assay, we demonstrated that our affinity biosensor could detect anti-SARS-CoV-2 antibodies. Using GNP-protein G to probe for bound anti-SARS-CoV-2 IgG antibodies, the affinity biosensor had increased impedance changes (∆Z_R_) with COVID-19 positive sera and minimal or decreased impedance changes with COVID-19 negative sera. Moreover, a ∆Z_R_ = 0 can be used as the threshold to classify the samples into positive or negative cases. This demonstrated that our affinity biosensor could discriminate between COVID-19 positive and negative sera which were further improved using polyvinyl alcohol as a blocking agent.

To design a biosensor that binds anti-SARS-CoV-2 antibody specifically, we needed to ensure that a homogeneous monolayer of the virus spike protein was attached to the surface of the electrode. While there are different methods to attach proteins to electrodes via primary amine surface functionalization^24^, aldehyde surface modification^25^, carboxylic acid functionalization^26^ or isothiocyanate linking^27^, successful attachment of protein onto the electrode surface is key to generating an efficient sensor. Regardless of the chosen surface chemistry, the S protein was attached using EDC/NHS standard coupling chemistry. We determined that carboxylic acid functionalization through succinic anhydride and then conjugation of protein S via EDC/NHS chemistry into the surface, gives the best surface coverage of S protein for this sensor. Cross-reactivity of the S protein was evaluated against several diseases (S. Babiuk et al., unpublished work). Using an ELISA, they showed that there is no cross reactivity to the spike protein and humans were previously infected with seasonal coronaviruses and other viral infections. There is cross reactivity between the spike antigens used from SARS-CoV-2 and SARS-Cov-1, the original SARS virus.

Recently, Rashed et al. presented a similar technique to detect COVID-19 using an impedance based detector^9^. Binding of antibody to the S protein can be measured in real time at a fixed frequency. However, the assay was very time sensitive because of the unstable binding between antibody and protein. We resolved this issue by adding gold nanoparticles conjugated to protein G which stabilized the binding of antibody to the S protein. Using in-house designed chips also allowed us to perform the measurements over a range of frequencies, thus identifying the best frequency at which one can easily distinguish the positive cases from the negative ones.

As of October 2021, there were 162 commercial immunoassays available that manually or automatically detect COVID-19 antibodies^28^. Several detect IgM, IgG, IgA, IgM/IgG or all three using ELISAs, microarrays or a biosensor (SD Biosensor, Inc). Commercial ELISA assays such as Affinity, Bio-Rad and Euroimmun, with 4 h assay time, 63% – 83% sensitivity is reported to indicate a COVID- 19 positive result^29^. The true positive rate of our impedance biosensor is 72% (13 correct results out of 18 tests) in 2 h, faster than an ELISA (4 h) with comparable sensitivity. In comparison with lateral flow assays which are as fast as 15 min, such as, RapidResponse (50–68% sensitivity), DeepBlue (53% sensitivity and Innovita (40% sensitivity), our designed biosensor performs with a higher accuracy. RapiGEN with 4 correct results out of 45 positive samples and Healgen with 12 false negatives out of 16 samples are outliers in this comparison^30^.

After running preliminary tests with designed sensor, we are now in the commercialization step. We are working on the development of an automatic device to control all the assay steps connected to a portable potentiostat which integrates all the functions within a hand-sized PCB board. The timing of electrode modification would be a challenge but can be solved with a larger scale production. The electrode fabrication and surface coating can be done all offline. As we tested, the prepared electrodes are pretty stable for 6 months. Gold nanoparticle-protein G conjugate works perfectly for 4 months. This biosensor could also be adapted to detect antibodies in other biofluids as it was demonstrated that anti-SARS-CoV-2 antibodies can also be detected in saliva^31^.

## Material and methods

### Materials

Gold (III) chloride trihydrate (HAuCl_4_), trisodium citrate dihydrate, *N*-hydroxysuccinimide (NHS), poly(ethylene glycol) 2-mercaptoethyl ether acetic acid, aminopropyltriethoxysilane (APTES), 3,3',5,5'-tetramethylbenzidine (TMB) substrate solution, Tris-base, bovine serum albumin (BSA), Tween 20, skim milk powder, polyvinylpyrrolidone (PVP-10) and polyvinylalcohol (PVA-61000) was supplied by Sigma-Aldrich (Oakville, Canada). Succinic anhydride 99% (ACROS Organics) was purchased from Fischer Scientific (Edmonton, Canada) and 1-ethyl-3-(3-dimethylaminopropyl)carbodiimide (EDC) 95% from AK Scientific (Union City, USA). Heterobifunctional polyethylene glycol (PEG) linker with 110 repeating PEG units containing a thiol group on one terminal and carboxylic acid on the other (SH-PEG_5000_-COOH) were purchased from Avanti Polar lipids, Inc. (Alabaster, USA). The Pierce™ recombinant protein G (Catalog number: 101200) and horseradish peroxidase (HRP) conjugated protein G (Catalog number: 101223), 96 well plates (BioLite) and Nunc MaxiSorp™ high protein-binding capacity 96 well ELISA plates were purchased from Thermo Fisher Scientific (Whitby, Canada). SARS-CoV-2 IgG developed against S1 (containing the receptor binding domain-RBD) was purchased from Creative Diagnostics (USA). Poly(dimethyl)siloxane (PDMS) was obtained from Dow Corning (Midland, USA). Ultrapure water (18.2 MΩ/cm) obtained from Millipore equipment (Milli-Q water) was used for samples preparation and washing. Impedance measurements were performed on Zurich Instruments MFIA controlled by the software LabOne® or the SP-200 potentiostat controlled by the EC-Lab® software package (Bio-Logic, TN, USA).

### Human serum samples

This study analyzed eighteen serum samples from nine SARS-CoV-2 infected patients and nine healthy donors. Blood samples were collected in Becton Dickinson SST tubes (containing silica clot activator) and centrifuged, according to manufacturer’s instructions to separate the serum. Human serum samples were categorized and verified as either SARS-CoV-2 antibody positive or negative by the Public Health Laboratory (Alberta Precision Laboratories, Edmonton, AB, Canada) via two different assays: Abbott ARCHITECT SARS-CoV-2 IgG (Nucleocapsid, Abbott, Chicago, USA) and DiaSorin LIAISON SARS-CoV-2 S1/S2 IgG (Spike, DiaSorin, Salugia, Italy).

### Cloning, expression and purification of the SARS-CoV-2 spike protein

The synthetic gene for SARS-CoV-2 spike protein (GenBank: QHD43416.1; 1273 amino acids) encodes for the amino acids 1–1213. The transmembrane domain region encompassing amino acids 1213–1237 and the cytoplasmic domain (amino acids 1237–1273)^32^ were removed to optimize expression. The synthetic spike protein gene was codon optimized for expression in baculovirus and was expressed cloned into a pABbee™-FH plasmid (containing an 8X-histidine affinity tag) by GenScript (Piscataway, USA). Recombinant baculovirus was generated by transfecting Sf9 cells (Expression Systems) with spike plasmids using the ProFold™-ER1 system (AB vector, San Diego, USA). Clones were screened by plaque purification. Master seed stocks were then prepared, characterized and used to generate working virus stocks. The working stocks were subcultured on Sf9 cells at 27 °C with shaking at 130 rpm for 120 h, and subsequently used to infect suspension cultures of TNI (Trichoplusia ni) cells (Expression Systems, Davis, USA) at a multiplicity of infection (MOI) of 5 to 10. Infected TNI cells were incubated at 27 °C with shaking at 130 rpm for 72 h for protein expression. The spike protein was purified by nickel-nitrilotriacetic acid (Ni–NTA) (Qiagen, Toronto, Canada) and the final product was confirmed to be functional by evaluation of the antigen using an in-house developed ELISA with positive and negative SARS-CoV2 sera.

### Transmission electron microscopy, ultraviolet–visible spectroscopy, dynamic/ electrophoretic light scattering, ellipsometry, atomic force microscopy

Bright field Transmission Electron Microscopy (TEM) was performed using a JEOL 2010 (LaB6 filament) electron microscope with an accelerating voltage of 200 keV. Samples were prepared by drop coating of nanoparticle dispersion onto a carbon-coated 200-mesh copper grids. Cryo-EM of GNP-PEG sample was provided by NanoCore (BC). Ultraviolet–visible spectroscopy (UV–Vis) was done using a Beckman Coulter DU^Ⓡ^730 spectrophotometer. Dynamic light scattering (DLS) measurements was completed on a Malvern Zetasizer Nano S series equipped with a 633 nm laser. Three measurements were performed for each sample at 25 °C. Zeta potential was obtained from electrophoretic light scattering (ELS) experiment on the same instrument. For ellipsometry a M-2000 V J.A. Woollam Ellipsometer was used to quantify the immobilization of S protein. The optical constant (ks, ns) and thickness of the pre-existing surface (silica with carboxylic acid groups) was obtained prior to immobilization of S protein and used for a substrate fitting model. Scanning wavelengths ranged from 370 to 730 nm applying a 65° incidental angle. The thickness of S protein layer was fitted through Cauchy models (An = 1.45, Bn = 0.01, Cn = 0.001)^33^. To prepare samples, the same surface modification protocol was followed on a thermal oxide coated silicon wafers but without electrodes. The binding of GNPs on IDE chip surface was characterized through atomic force microscopy (AFM) on a diMultiMode V SPM, Veeco Instrument. Samples were scanned at a scan rate of 0.1 Hz using tapping mode. AFM images were processed and analyzed using Nanoscope software.

As-synthesized gold nanoparticles and GNP-protein G conjugates were analyzed by the Metals/Minerals (metallomics) assay performed by inductively coupled plasma-mass-spectrometry (ICP-MS) at The Metabolomics Innovation Centre (TMIC) (Edmonton, Canada).

### Synthesis and functionalization of gold nanoparticles

Gold nanoparticles (GNP) were synthesized following a modified Turkevich method^34^. A heterobifunctional polyethylene glycol (PEG) linker with 110 repeating PEG units containing a thiol group on one terminal and carboxylic acid on the other (SH-PEG_5000_-COOH) was used to modify the surface of the GNPs to prepare them for protein conjugation. Briefly, 50 mL 1 mM gold hydrochlorate was heated to boiling. Then 5 mL 38.8 mM trisodium citrate was added rapidly while stirring. After 10 min, heat was removed and the mixture stirred for another 20 min to cool down. To prepare stable carboxylated GNPs, 11 µL 0.1 mM SH-PEG_5000_-COOH (prepared in Mili-Q water) was mixed with 1 mL GNP-citrate at a 1000:1 gold to linker molar ratio^35^. The GNP-COOH reaction mixture was incubated for 12 h at RT, centrifuged and washed three times with Milli-Q water at 15,000×*g* for 45 min at 4 °C. The washed GNP-COOH conjugates were suspended in Milli-Q water and were stored at 4 °C.

### Conjugation of GNP-COOH with protein G

A mixture of 30 mg EDC and 36 mg NHS was prepared in 1 mL of 10 mM MES buffer pH 5.5. 10 µL of 13 nm GNP-PEG (OD 50 in water, measured in 1 cm cuvette) was mixed with 10 µL of EDC/NHS solution and incubated for 30 min at RT. Then, 1 mL of 10 mM PBS, pH 7.4 containing 0.05% Tween 20 (0.05% PBST) was added and vortex thoroughly. The supernatant was separated and removed by centrifugation at 13,000×*g* for 1 h. 10 µL of 1 mg/mL protein G (in PBS) was added, sonicated for 10 s in a water bath and incubated for 3 h at RT. The final GNP-proteinG conjugate was washed by adding 1 mL PBST, vortex and centrifuged at 13,000×*g* for 1 h at 4 °C. GNP-proteinG was re-suspended in 50 µL 10 mM PBS with 1% BSA and stored at 4 °C.

### ELISA assay for detection of anti-SARS-CoV-2 spike protein antibodies

The Nunc MaxiSorp™ high protein-binding capacity 96 well ELISA plates were coated in duplicate with 250 ng or 150 ng per well of baculovirus-purified SARS-CoV-2 S protein (prepared in 10 mM PBS, pH 7.4) at 4 °C overnight. The next day, wells were washed four times with 0.05% PBST and blocked with 0.05% PBST containing 3% skim milk powder for 2 h at RT. The wells were then incubated with either commercial SARS-CoV-2 IgG developed against S1 (0.5 – 1000 ng/mL) or serum samples from COVID-19 patients or healthy volunteers diluted 1:10 in 0.05% PBST containing 1% milk for 1 h at 37 °C. Two blank wells (containing 1% milk only and no serum) were included as negative controls on each plate. After washing five times with 25 mM Tris-buffered saline containing 0.2% Tween 20 (pH 7.4; 1X TBST), HRP-conjugated PrG diluted 1 in 2,000 (at final concentration of 500 ng/mL) in 50 mM phosphate buffer (pH 6) was added and incubated for 1 h at RT. Wells were then washed five times with 1X TBST and dried by vigorous tapping of plates on Kimwipes. 100 µL of TMB substrate solution was added and the reaction was stopped after 15 min by the addition of 2 N sulfuric acid. The A_450_ was measured in a BioTek ELX800 Microplate reader. The coefficient of determination (R^2^) of the A_450_ data was calculated using Microsoft Excel.

### Chip fabrication, IDE cleaning, pre-treatment and functionalization with S protein

The in-house sensor chip consists of eight IDEs fabricated on a rectangular glass slide (40 × 30 × 1 mm^3^). A PDMS mask was used to prepare eight separat, individual wells (3 mm^2^) to fit the IDEs. Each IDE consists of 750 gold microelectrodes arranged in parallel with length, width, thickness and a gap of 2.7 mm, 2 µm, 60 nm and 2 µm, respectively.

Prior to functionalization, the IDEs (in glass petri dishes) were cleaned by sequentially sonicating for 3 min each in acetone, isopropanol, and Milli-Q water and then drying under a stream of nitrogen. The chips were then exposed to oxygen plasma (100 sccm O_2_, 150 mT pressure, 150 W RF) for 40 s to hydroxylate the SiO_2_ surface. The cleaned chips were immediately silanized by immersing in a 5% APTES (in 100% ethanol) for 3 h at 4 °C. Then the chips were washed sequentially with absolute ethanol, isopropanol, and Milli-Q water to remove the unreacted APTES followed by drying under a stream of nitrogen. The chips were then immersed in a 10% succinic anhydride solution in 0.1 M sodium acetate pH 4.5 for 12 h of shaking, washed 5 times each with 10 mM PBS and then Milli-Q water and finally dried under an air flow.

The chips were chemically activated with EDC/NHS to prepare for S protein binding. 40 µL of a fresh solution of 30 mg EDC and 36 mg NHS in 1 mL of 10 mM MES buffer (pH 5.5) was added to each well and incubated for 15 min at RT. The solution was removed and wells washed once with PBS. 40 µL of S protein (200 µg/mL in 10 mM PBS, pH 8) was added to each well and incubated at 37 °C for 1 h. The wells were aspirated and washed three times each with PBS, then Milli-Q water.

### Detection of SARS‐CoV‐2 IgG using GNP-protein G conjugate

After completing the functionalization of the IDEs with S protein, aspiration and washing with PBS and Milli-Q water, unoccupied surface spots were blocked with 40 µL of different blocking solutions (1% BSA, 3% PVP-10, 3% milk and 1% PVA-61000) overnight at RT. The wells were aspirated and washed three times each with PBS, then Milli-Q water. Then, the patient's serum samples (containing SARS‐CoV‐2 IgG) or serum from healthy volunteers were diluted 10 times with 1% PVA in PBS and incubated on the chip for 1 h at 37 °C. Wells were washed two times with 10 mM PBS containing 0.2% Tween 20 and then GNP-protein G (diluted in PBS) was incubated on the chip for 1 h at RT protected from light. Wells were aspirated and washed 3 times each with 10 mM, then 10 µM PBS.

### Impedance measurement and analysis

Each sensor chip consists of eight IDEs allowing one to perform eight simultaneous impedance measurements. An input signal of 10 mV (with zero DC bias) was applied to the IDE sensor. The output signal, the impedance spectrum of each 8 electrodes, was measured from 10 to 1000 kHz. The total impedance of the equivalent circuit is represented in Eq. (1) below where Z is the total impedance of the system, R_s_ is the resistance of the solution, Z_g_ is the extra impedance imposed by the bound GNPs, Z_dl_ is the impedance of the double layer, C_geo_ is the dielectric (geometric) capacitance of the system, and Z_par_ is the parasitic impedance of the substrate. The values j and ω are the imaginary number and angular frequency, respectively.

1 $$\mathrm{Z}= (\mathrm{R}_{\mathrm{s}} +\mathrm{ Z}_{\mathrm{g}} + 2\mathrm{Z}_{\mathrm{dl}})|| 1\mathrm{ j}\omega C_{geo} ||\mathrm{Z}_{\mathrm{par}}$$

Z_dl_ contribution arises from the formation of the ionic double layer at the charged surface. Under measurement conditions, a double layer of ions forms at the interface of any charged surface, which functions as a capacitor and can be represented as C_dl_. This double layer behaves like a capacitor and contributes an impedance Z_dl_ to the system (one for each electrode finger in the pair).

The impedance measurement was done after every single step of the surface modification, addition of serum, and the GNP-protein G treatment by washing wells and pipetting 50 µL 10 µM PBS in each well. After each step, the relative ∆Z_R_ was calculated by subtracting the value of the impedance at the previous step from the current one and dividing by the value of the former. For example, the relative ∆Z_R_ for the last step of detection of SARS‐CoV‐2 IgG using GNP-protein G conjugate is defined in Eq. (2) below where Z_serum_ and Z_gold_ stand for the absolute value of the total impedance |Z| after the addition of the serum and the GNP-protein G (gold), respectively.

2 $$\Delta {\mathrm{Z}}_{\mathrm{R}}= ({\mathrm{Z}}_{\mathrm{gold}} - {\mathrm{Z}}_{\mathrm{serum}})/{\mathrm{Z}}_{\mathrm{serum}}$$

∆Z_R_ emphasizes changes introduced at each step due to the given additive, i.e., serum, GNP-proteinG, etc., and provides us with a consistent and uniform yardstick to distinguish positive cases from the negative ones. To ensure that the collected data are reliable and the chip is functioning well, the standard deviation of the relative impedance over the range of the frequency is calculated and those with values less than 1% have been dropped from the analysis.

### Ethics declarations

This work was conducted in accordance with approval and guidance set out by the research ethics boards at University of Calgary (Study ID REB20-0516) and University of Alberta (Study IDs Pro00099818, Pro00101916, and Pro00109215). All study participants were above the age of 18 and provided full informed consent to participate, as per guidance by research ethics boards.

## Supplementary Information

Supplementary Figures.
