# Supplementary material for: A gold nanoparticle-protein G electrochemical affinity biosensor for the detection of SARS-CoV-2 antibodies: a surface modification approach
Source: Sci Rep. 2022 Jul 27;12:12850. doi: 10.1038/s41598-022-17219-7 (PMC9328775; doi:10.1038/s41598-022-17219-7)
Supplement: Supplementary file 1 — Supplementary Figures. [file 41598_2022_17219_MOESM1_ESM.docx]

Supplementary Materials for

**A gold nanoparticle-protein G electrochemical affinity biosensor for the detection of SARS-CoV-2 antibodies – a surface modification approach**

Yeganeh Khaniani, Yuhao Ma, Mahdi Ghadiri, Jie Zeng, David Wishart, Shawn Babiuk, Carmen Charlton, Jamil N. Kanji, Jie Chen*

*Corresponding author. Email: jc@ualberta.ca

**This PDF file includes:**

Figs. S1 to S3


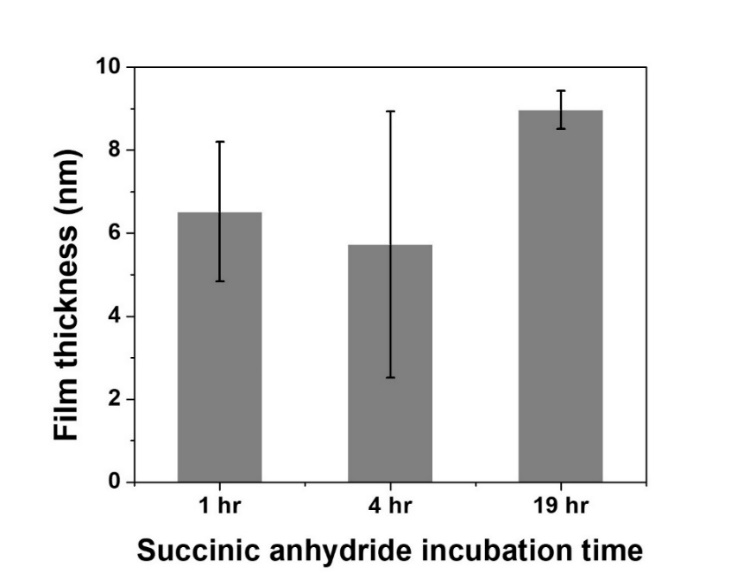


Fig. S1.

**Thickness of S protein layer measured by ellipsometry.** The longest incubation time with succinic anhydride solution resulted in the thickest layer. Data represent mean ±1 SD; n=3.


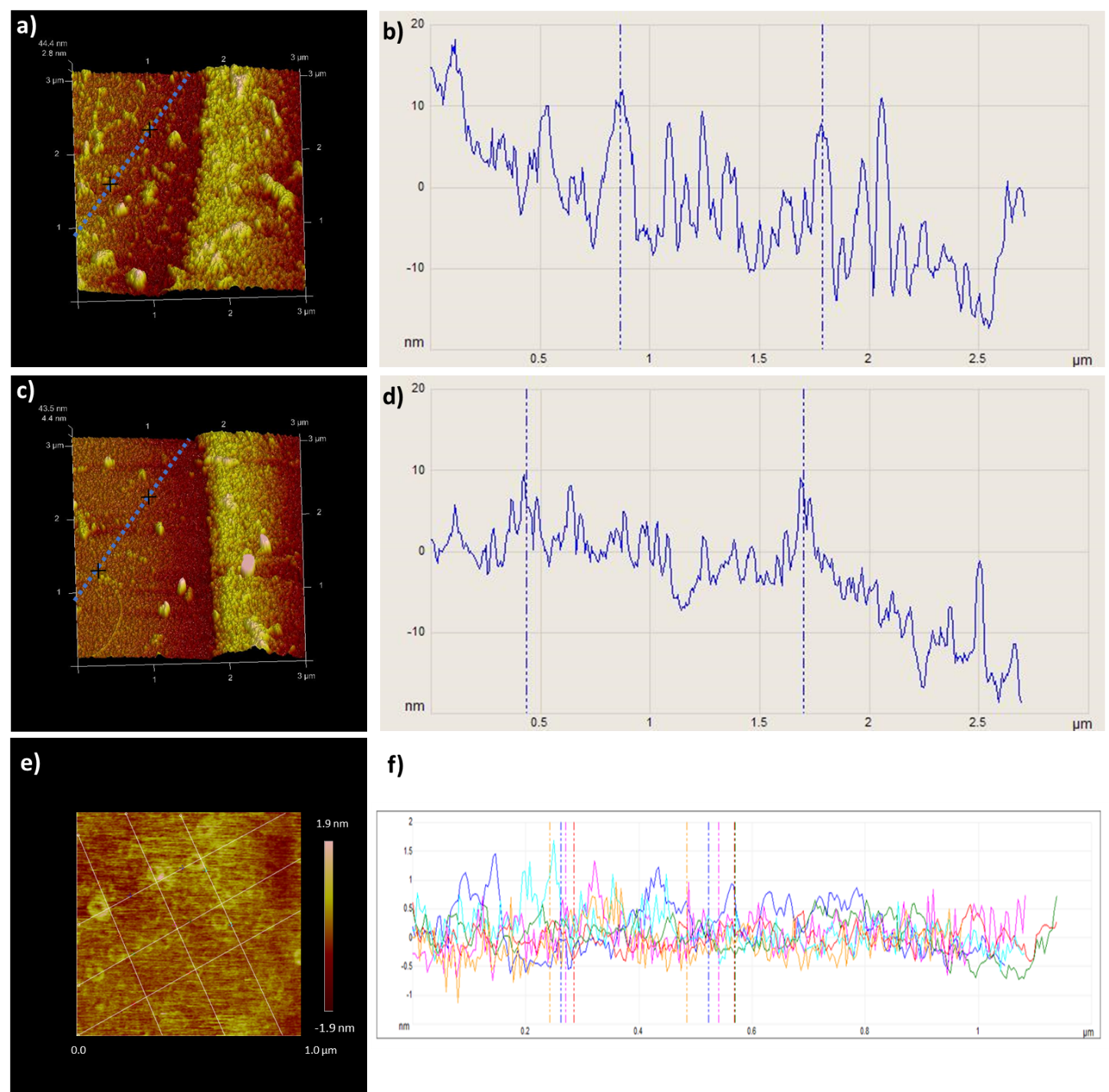


Fig. S2.

**Atomic force microscopy images of the biosensor silica surface after incubation with GNP-Protein G**. Images of gap between interdigitated electrode (IDE) incubated with COVID-19 positive (a,b) or negative (c,d) serum sample. The electrode without S protein coating (e, f).


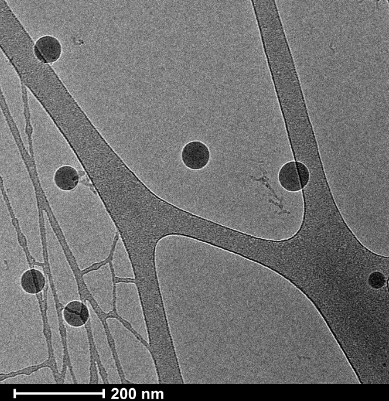


Fig. S3.

CryoEM image of gold nanoparticles decorated with PEG chains. 25 nm particles are presented in this sample in compared to 12 nm particles in as-made gold nanoparticles.
